# The role of comparison processes in maintenance goals: Evidence from the health and relationship domains

**DOI:** 10.1111/aphw.70133

**Published:** 2026-03-10

**Authors:** Yael Ecker, Kathi Diel, Wilhelm Hofmann, Christian Unkelbach, Roland Imhoff

**Affiliations:** ^1^ Social Cognition Center Cologne University of Cologne Cologne Germany; ^2^ Department of Psychology Saarland University Saarbrücken Germany; ^3^ Department of Psychology Ruhr University Bochum Bochum Germany; ^4^ Department of Legal and Social Psychology Johannes Gutenberg University Mainz Mainz Germany

**Keywords:** goal pursuit, maintenance goals, motivation, self‐comparisons, social comparisons

## Abstract

This research examined how mental comparisons impact maintenance striving—the ongoing care for one's valued current state—with implications for understanding daily struggles to maintain personal health and well‐being. Following the ternary goal model, we hypothesized that (H1) maintenance goals, differently from progress goals, are not motivated by upward social comparisons, which reduce appreciation for current states, and that (H2) maintenance goals are motivated by upward self‐comparisons, which increase appreciation for current states. We tested our hypotheses in five preregistered studies (*N* = 2435) employing experimental designs in health (Studies 1 and 2) and relationship (Studies 3 and 4) domains, experience sampling methodology (Study 4), and behavioral measures (Study 5). Hypotheses were confirmed across all studies: Maintenance motivation was consistently higher following upward self‐comparisons and lower following upward social comparisons. Mental comparisons influenced maintenance goals through changes in appreciation for one's current state, with Study 1 providing direct evidence that these effects differ for progress goals. By triangulating findings from experience sampling, controlled experiments, and behavioral measures, we gain a robust understanding of this psychological phenomenon. These findings advance goal theory and provide practical insights for developing interventions that support long‐term health behavior maintenance, relationship satisfaction, and overall well‐being in applied settings.

## INTRODUCTION

The ancient Jewish proverb “be a tail unto lions, and not a head unto foxes” reflects the belief that exposure to superior others leads to self‐improvement—a principle that finds strong support in modern psychological research. Upward social comparisons to better‐off others provide both practical tools and motivational inspiration for self‐improvement (Aspinwall et al., 2002; Taylor & Lobel, 1989; Wheeler, 1966). However, this well‐documented effect may not extend to all types of goals. In the current research, we challenge this consensual view by examining comparison processes in the context of maintenance goals—goals that guide ongoing care for valued current states such as health, relationships, and personal well‐being. We propose that while better‐off others inspire improvement goals, they are unlikely to motivate one to maintain what one already has. Instead, we argue that upward self‐comparisons more effectively motivate maintenance goals. In making these claims, we rely on a ternary goal framework of goal types (Ecker, 2024; Ecker & Moors, 2026), which provides novel predictions about the relationship between comparisons and maintenance goal pursuit. By confirming these predictions across multiple domains and methodologies, we lay out evidence for the unique nature of maintenance goals.

## MAINTENANCE AS A DISTINCT GOAL TYPE

Following a ternary goal distinction, people may pursue three types of goals: progress, protection, and maintenance goals (Ecker, 2024; Ecker et al., 2022; Ecker et al., 2023). Progress goals guide attempts to change the current state, and protection goals guide attempts to reduce threats to the current state. Conversely, maintenance goals guide attempts to regularly maintain the current state of affairs in the absence of threats to be reduced or further rewards to be won (Ecker & Gilead, 2018). For example, in the financial domain, a progress goal might involve increasing yearly revenue, while a protection goal might involve responding to risks that threaten losses. In contrast, a maintenance goal would guide routine care for financial assets when threat levels are stable and unchanging. Maintenance goals are presumably most common during periods of stability and safety, when existing threats cannot be further reduced and progress opportunities are limited.

Maintenance goals satisfy the routine needs of stable positive states, whether it is one's relationship (e.g. by regularly spending time together), one's health (e.g. by cooking healthy meals at home), or one's household (e.g. by completing weekly house chores). They reflect ongoing care for existing states without further improving or adding to them. Such daily activities—meeting friends, date nights with partners, tucking children into bed, exercising regularly, and cooking healthy meals—are prevalent and essential to personal well‐being. Research that tested the self‐reported prevalence of maintenance compared with progress and protection goals found that maintenance accounted for about 40% of personal goals in varied domains (Ebner et al., 2006; Ecker et al., 2022).

According to the ternary goal model (Ecker, 2024), people's regular efforts to maintain their relationships, health, and possessions operate through a goal mechanism that is inherently distinct from those of progress and protection. As a result, maintenance is motivated by different factors than progress and protection (Ecker et al., 2022, 2023; Rothman, 2000; Rothman et al., 2004). Most importantly for this paper, this distinct mechanism carries unique implications for how comparisons influence maintenance motivation—implications that have thus far remained unexplored.

This represents a critical gap for both theoretical and practical reasons. Theoretically, if maintenance goals respond differently than progress goals to the same input, this would provide compelling evidence that maintenance operates through a distinct psychological mechanism. Practically, health interventions frequently employ social comparison strategies (e.g. Brent et al., 2015; Mahler et al., 2010; for a comprehensive meta‐analysis, see Hoppen et al., 2025) to motivate behavior change, but it remains unknown whether these same strategies effectively support long‐term maintenance. Given that maintenance represents one of health psychology's most persistent challenges (Rothman, 2000), understanding which comparison processes support versus undermine maintenance motivation is essential for designing effective interventions.

## EFFECTS OF COMPARISON WITH OTHERS ON MOTIVATION

Upward comparisons with better‐off others can play an important role in instigating progress goals. For instance, if Jill wants to become a successful scientist, she may draw inspiration from acclaimed scientists in her social environment (an upward social comparison). When comparison standards are perceived as attainable and relevant, research suggests that upward social comparisons inspire greater investment in approaching desired outcomes (Aspinwall et al., 2002; Diel et al., 2021; Diel & Hofmann, 2019; Lockwood & Kunda, 1997; Taylor & Lobel, 1989; Wheeler, 1966). However, when standards seem extreme or out of reach, upward comparisons can lead to discouragement rather than increased goal pursuit (Diel et al., 2021; Diel & Hofmann, 2019). The present research therefore centers on attainable comparisons with ordinary others in relatable situations—contexts in which upward social comparisons tend to have motivating effects on progress goals.

Yet upward comparisons with attainable standards, while motivating progress, reduce evaluations of one's current state (Aspinwall et al., 2002; Buunk et al., 1990; Wood, 1989)—a cost especially relevant to maintenance goals. Because maintenance goals reflect the pursuit of a continued positive state, maintenance motivation is primarily influenced by the value one assigns to their current situation (Ecker et al., 2022; Ecker & Gilead, 2018; Rothman, 2000; Rothman et al., 2004). The more one appreciates what they have, the more they mobilize to sustain it. When Jill considers a successful scientist, her progress goal gains greater value and salience. However, if Jill aspired to maintain her current success level, thinking of a superior scientist may diminish her appreciation for her own record, thereby reducing her motivation to maintain it. Conversely, thinking of an unsuccessful scientist may increase Jill's sense of appreciation and thereby boost her motivation to maintain.

Accordingly, our first prediction is that *upward social comparisons will be less effective than downward social comparisons at motivating maintenance goals*. Confirming this prediction would suggest that social comparisons influence maintenance goals differently than progress goals. This insight leads to a crucial next question: What kinds of comparisons *do* effectively motivate maintenance? To address this, we turn from social comparisons to self‐comparisons.

## EFFECTS OF SELF‐COMPARISONS ON MOTIVATION

Comparisons with others are not the only form of comparison that people are strongly inclined to make. A prominent alternative is comparisons with one's own self‐representations. While downward comparisons with past selves serve a self‐enhancement function (e.g. Baldwin et al., 2023; Wilson & Ross, 2001), upward comparisons with future selves direct desired changes and motivate individuals to realize desired visions of future selves (Hoyle & Sherrill, 2006; Markus & Nurius, 1986; Oyserman et al., 2004). Additionally, comparisons with positive possible selves increase one's perceived self‐worth and appreciation for the current state (Bak, 2015; Loveday et al., 2018).

Why might upward self‐comparisons enhance appreciation? Comparison process models (Bless & Schwarz, 2010; Mussweiler & Strack, 1999) suggest that when comparing with highly similar targets—such as one's own possible selves—comparison information is more likely to assimilate into current self‐evaluations. When the comparison standard is positive, this assimilation process may enhance how positively one evaluates oneself on the relevant dimension (e.g. “I am like my healthy ideal self”). Such enhanced self‐evaluations may, in turn, foster greater appreciation for one's current state.

The boosting effect of upward self‐comparisons on appreciation makes them strong candidates for inspiring maintenance goals. For instance, when Jill envisions her ideal healthy self, she may feel boosted appreciation for her current health, leading to greater motivation to maintain her existing healthy lifestyle. Conversely, social comparisons involve fundamentally different individuals, reducing perceived similarity and making contrast effects more likely (Mussweiler & Strack, 1999), Such contrast effects, in turn, are likely to reduce rather than enhance appreciation for one's current state.

An additional consideration is that self‐comparisons inherently involve highly attainable standards compared with social comparisons. When comparing with one's self, concerns about whether the standard is achievable are minimized. This difference in inherent attainability may make such comparison generally more motivating in addition to the specific motivational boost to maintenance goals.

While upward self‐comparisons lead to greater appreciation, downward self‐comparisons likely increase an emotional experience of threat (Smith, 2000). For instance, envisioning one's own health deteriorating rapidly is likely a threatening experience. According to the ternary goal model, threat propels protection but not maintenance goals (Ecker, 2024). Jill may therefore feel propelled to schedule medical screenings or take preventive medications, but she would not invest more in regularly maintaining her current healthy lifestyle. Accordingly, our second prediction is that *upward self‐comparisons will motivate maintenance more than downward self‐comparisons*.

## THE CURRENT RESEARCH

Across five studies, we manipulated comparison direction (downward vs. upward) and tested its effect on maintenance striving. Studies 1 to 3 used simulated scenarios to test the influence of social comparisons on motivation for maintenance in health (Studies 1 and 2) and relationships (Study 3). Study 4 brought maintenance striving to everyday life in a 6‐day longitudinal study with 11 measurements, manipulating participants' self‐comparison direction and measuring their daily investments in maintaining their romantic relationships. Finally, Study 5 provided a behavioral measure by having participants play a house maintenance game while viewing either better and worse houses of other players (social comparisons) or better and worse houses they could have (self‐comparisons). The time and effort participants invested in the maintenance task served as our behavioral measure of maintenance motivation.

## TRANSPARENCY STATEMENT

We preregistered our predictions in all studies (see preregistration links in the study intros). Across all studies, we report how we determined our sample size, all data exclusions (if any), all manipulations, and all relevant measures. All materials pertaining to this project are fully available on the OSF (see link: https://osf.io/agvkh). Additional studies that are not included in the paper for considerations of length and fluency are also openly available on the OSF. In Figures S3 and S4, we report a meta‐analysis that includes all excluded studies conducted in this research line in order to avoid a file‐drawer effect.

## STUDY 1

We tested whether upward (vs. downward) social comparisons would boost progress motivation but not maintenance motivation in health. We predicted and preregistered (https://aspredicted.org/blind.php?x=w3xv9y) that upward other comparisons would decrease appreciation for one's current state and that this decrease would mediate subsequent decreases in maintenance motivation.

### Method

We planned to recruit 200 participants per condition and invited 400 participants on Prolific residing in the United States, United Kingdom, or Ireland, ages 25–75. Data were collected in January 2021. The final sample included 402 participants (65% women, *M*_age_ = 41.19, *SD* = 12.65), providing 80% power to detect a two‐way within‐between interaction with *η* ^2^ *p* = .02, as determined using G*Power (Faul et al., 2007). We randomly assigned participants to report either health aspects they wanted to improve (progress goal) or maintain (maintenance goal). All participants considered scenarios about healthier (upward comparison) and less healthy (downward comparison) neighbors in randomized order. The comparison scenarios were deliberately designed to describe ordinary neighbors with ordinary health situations to ensure that all comparison standards would be perceived as attainable. After each scenario, participants reported their agreement with two motivation statements (“Thinking about this scenario motivates me to maintain/improve my current health” and “Thinking about this scenario encourages me to put effort in maintaining/improving my current health”) and one appreciation statement (“Thinking about this scenario helps me appreciate my current health”) on 7‐point Likert scales.^1^

### Results

**FIGURE 1:**
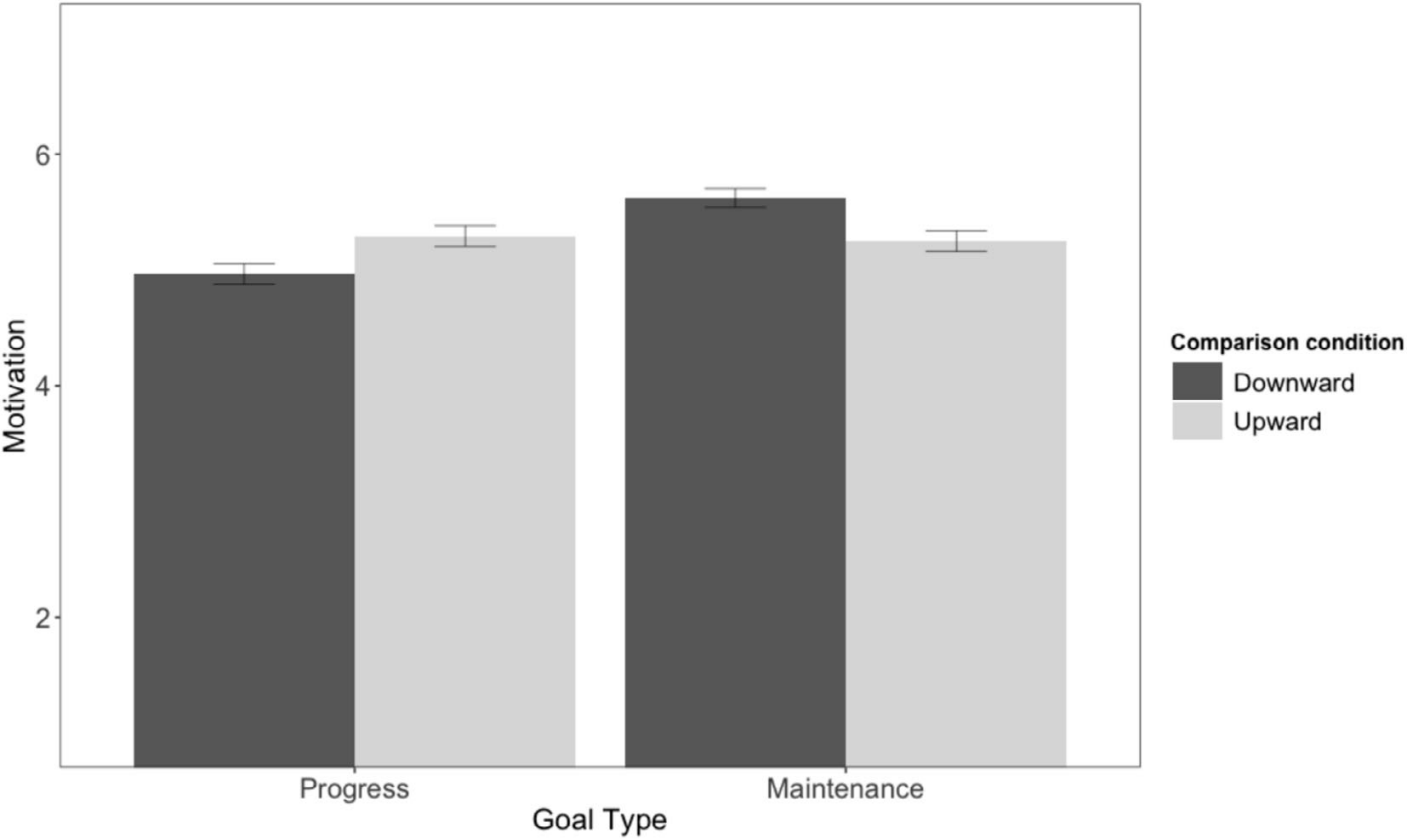
Study 1: Motivation by goal type (maintenance, progress) and social comparison direction (downward, upward) in the health domain. *Note*: Error bars indicate standard errors. Motivation measured on a 7‐point Likert scale.

As preregistered, we averaged the two motivation items into a composite motivation score (*r* = .84 and .83 across presentation orders). A 2 (goal type) × 2 (comparison direction) ANOVA on the composite motivation score yielded the predicted interaction, *F*(1, 400) = 33.89, *p* < .001, *η* ^2^ *p* = 0.08. Health progress motivation was higher after upward than downward social comparisons, *t*(203) = 3.63, *p* < .001, whereas health maintenance motivation was higher after downward than upward comparisons, *t*(197) = −4.79, *p* < .001 (see Figure 1). Additionally, upward social comparisons significantly decreased appreciation for current health (*M* = 4.02 vs. 5.47), *F*(1, 400) = 349.04, *p* < .001, *η* ^2^ *p* = .47. We used the MLMED macro for SPSS (Hayes & Rockwood, 2020) to calculate a 2‐1‐1 mediation, with comparison direction as a Level 2 predictor, appreciation as Level 1 mediator, and investment in maintenance as the Level 1 dependent variable. This mediation analysis revealed that decreased appreciation fully mediated the negative effect of upward comparisons on maintenance motivation (indirect effect = −.13, 95% CI [−0.20, −0.06], *z* = −3.56, *p* < .001).

### Discussion

Study 1 found a strikingly distinct influence of social comparisons on the motivation to maintain one's health compared with the motivation to improve health. In line with the existing literature, approach motivation was greater after upward (vs. downward) other comparisons. In contrast, maintenance motivation was lower after upward other comparison, and this decrease in motivation was fully mediated by decreases in appreciation for the current state. Indeed, it seems that maintenance goals—which are uniquely sensitive to the value of the current state—are not effectively motivated by upward other comparisons.

## STUDY 2

In Study 2, we zoom in on people's personal health maintenance goals and extend our investigation to test the impact of both other and self‐comparisons. We preregistered (link: https://aspredicted.org/JHC_VF6) the prediction that comparison direction and comparison type will interact such that upward (vs. downward) self‐comparisons will boost maintenance motivation more than upward (vs. downward) other comparisons.

### Method

We recruited 500 UK residents (ages 30–75) on Prolific in May 2023. Participants first reported two health maintenance activities and were then asked whether they viewed these as maintenance or improvement activities. We preregistered keeping only participants who view their activities as maintenance, which resulted in a final sample of 210 participants (47% women; *M*_age_ = 48.94, *SD* = 11.78), providing 80% power to detect *η* ^2^ *p* = .03 as determined using G*Power (Faul et al., 2007). Participants were randomly assigned to either social comparison or self‐comparison conditions. All participants viewed both downward and upward comparison scenarios in random order. Social comparison scenarios described very healthy or unhealthy neighbors—with all scenarios depicting attainable situations involving ordinary individuals—while self‐comparison scenarios instructed participants to think of a version of themselves as healthier or less healthy than ever before. After each scenario, participants rated their appreciation for current health, perceived threat, and motivation to maintain their health on 7‐point scales.

### Results

**FIGURE 2:**
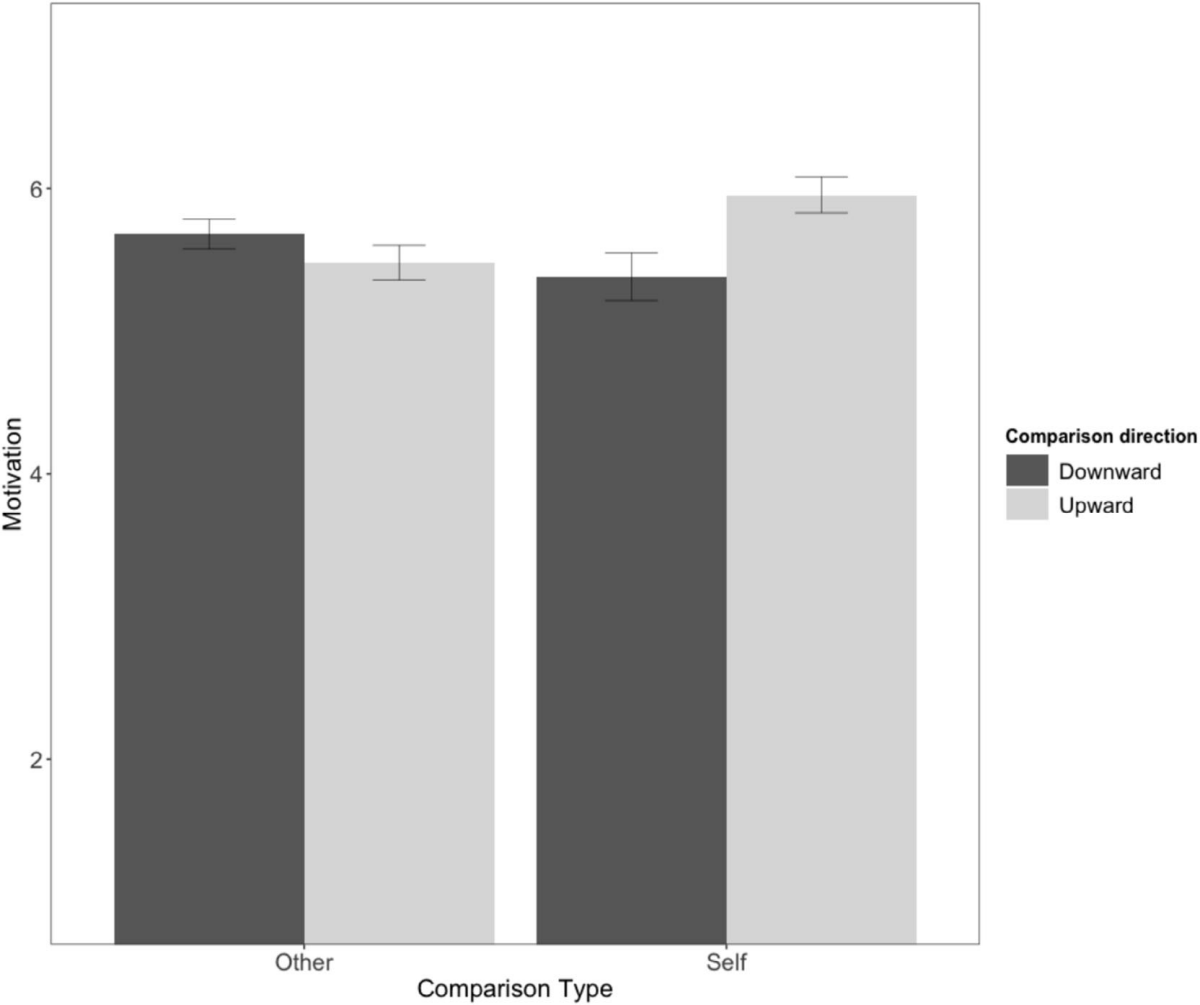
Study 2: Maintenance motivation by comparison type and comparison direction. *Note*: Error bars indicate standard errors. Motivation measured on a 7‐point Likert scale.

As preregistered, we averaged the two motivation items into a composite maintenance motivation score (*r* = .94 and .95 across presentation orders). A 2 (comparison type: self vs. social) × 2 (comparison direction: downward vs. upward) ANOVA on maintenance motivation revealed the predicted interaction, *F*(1, 208) = 17.55, *p* < .001, *η* ^2^ *p* = .08. Upward self‐comparisons increased maintenance motivation more than downward self‐comparisons, *t*(101) = 3.47, *p* < .001, *d*z = 0.38, while upward social comparisons decreased maintenance motivation relative to downward social comparisons, *t*(107) = −2.25, *p* = .026, *d*z = 0.17 (see Figure 2; see Table S1 for zero‐order correlations).

Exploratory mediation analyses revealed that appreciation mediated these effects. Upward self‐comparisons increased appreciation (*M* = 5.89 vs. 4.93), *t*(101) = 3.01, *p* = .003, while upward social comparisons decreased appreciation (*M* = 4.78 vs. 5.82), *t*(107) = −9.55, *p* < .001. Appreciation significantly mediated maintenance motivation for both self‐comparisons (indirect effect = .20, 95% CI [0.05, 0.35]) and social comparisons (indirect effect = −.11, 95% CI [−0.21, −0.03]). Although comparisons also affected perceived threat—with upward self‐comparisons reducing threat (*M* = 2.47 vs. 3.49), *t*(101) = −5.35, *p* < .001, and upward social comparisons increasing threat (*M* = 2.98 vs. 2.25), *t*(107) = 4.90, *p* < .001—threat did not significantly mediate maintenance motivation in either condition (*p*s > .09). These results confirm that maintenance motivation operates specifically through appreciation for one's current state, not through threat‐related mechanisms.

### Discussion

Study 2 replicated and extended Study 1's findings. Upward self‐comparisons enhanced maintenance motivation by increasing appreciation for one's current health, while upward social comparisons undermined maintenance motivation by reducing such appreciation. The lack of threat‐based mediation is consistent with our theoretical prediction that maintenance motivation operates through appreciation‐based rather than threat‐based mechanisms.

## STUDY 3: RELATIONSHIP MAINTENANCE AND GOAL SHIFTING

Next, we tested our predictions in the relationship domain. This domain is particularly important as maintenance goals are most prevalent in relationships compared with other life domains (Ecker et al., 2022). Moreover, confirming our prediction across both health and relationship contexts would strengthen confidence that our findings reflect general properties of maintenance motivation rather than domain‐specific effects.

Participants reported aspects they wanted to maintain in their romantic relationships, then viewed comparisons of better or worse romantic couples (social comparisons) or better or worse versions of their own relationship (self‐comparisons). We predicted and preregistered (https://aspredicted.org/JQN_LZT) that the upward (vs. downward) self‐comparisons would boost the motivation to maintain the current state of the relationship more than upward (vs. downward) other comparisons.

Crucially, we also measured *goal shifting*—whether participants switched from wanting to maintain to wanting to improve their relationship—to test an alternative explanation for our predicted effects. Specifically, decreased maintenance motivation after upward social comparisons or downward self‐comparisons might reflect participants shifting to progress goals rather than simply reduced motivation to maintain. This measure allowed us to test whether goal shifting, alongside appreciation, mediated comparison effects on maintenance motivation.

### Method

We recruited 450 UK residents on Prolific in May 2023. Participants reported two relationship aspects they wanted to maintain and confirmed they viewed these as maintenance (not improvement) goals. This exclusion criterion resulted in 329 participants (50% women; *M*_age_ = 43.57, *SD* = 12.30), providing 80% power to detect *η* ^2^ *p* = 0.025 as determined using G*Power (Faul et al., 2007). Participants were randomly assigned to view either social comparisons (better/worse neighboring couples) or self‐comparisons (better/worse versions of their own relationship) in random order. After each scenario, participants rated their appreciation, motivation to maintain their relationship, and whether their goal shifted from maintenance to improvement. The full procedure is presented in the Supporting Information.

### Results

**FIGURE 3:**
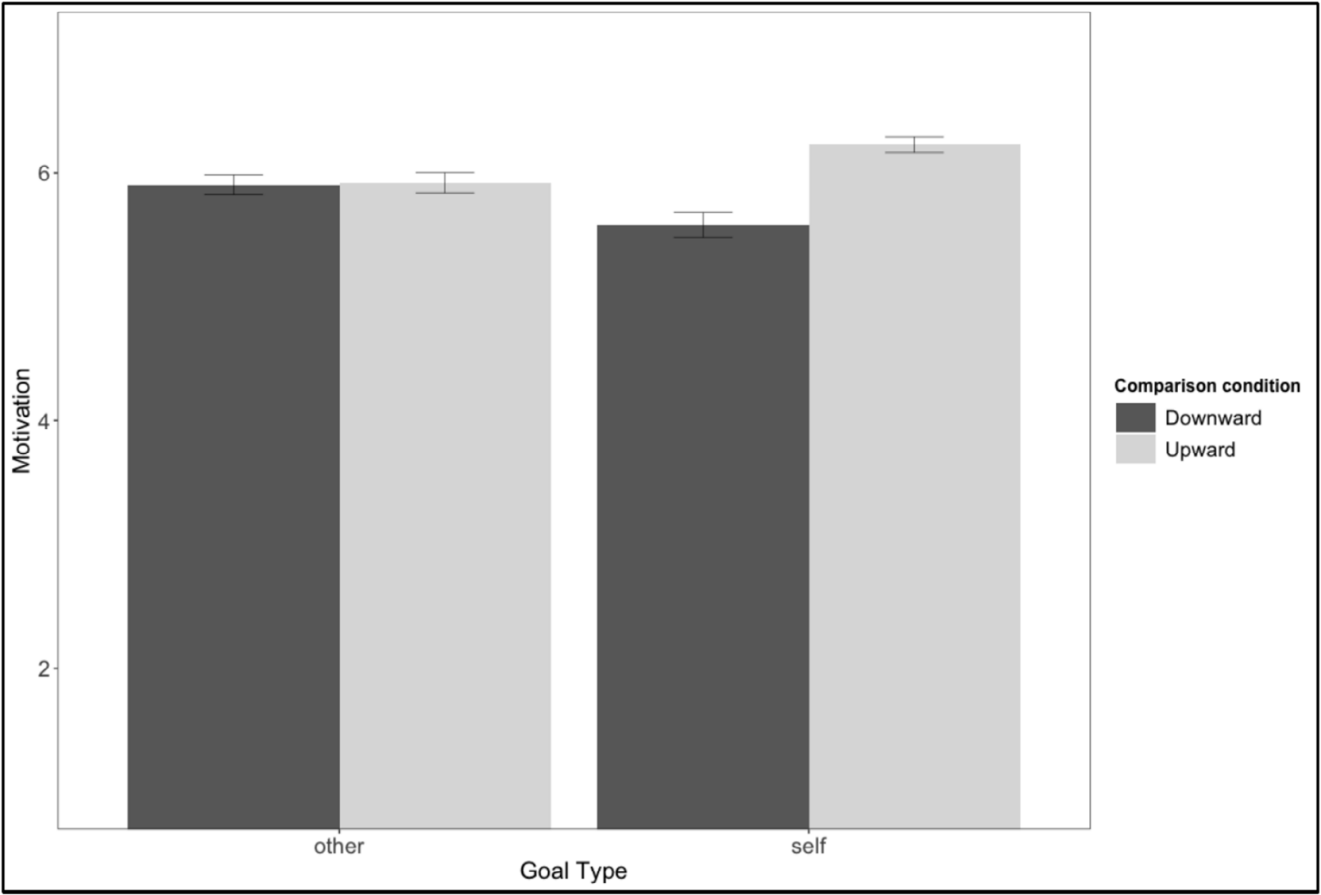
Study 3: Relationship maintenance motivation by goal type and comparison direction. *Note*: Error bars indicate standard errors. Motivation measured on a 7‐point Likert scale.

As preregistered, we averaged the two motivation items into a composite maintenance motivation score (*r* = .76 and .83 across presentation orders). Maintenance motivation showed the predicted interaction, *F*(1, 327) = 30.43, *p* < .001, *η* ^2^ *p* = .09. Upward self‐comparisons increased maintenance motivation more than downward self‐comparisons, *t*(167) = 6.40, *p* < .001, *d*z = 0.62, while social comparisons had no effect, *t*(160) = −0.05, *p* = .96 (see Figure 3; see Table S2 for zero‐order correlations). We then explored whether this effect persisted even when restricting analyses to participants who never shifted goals. We again found a significant effect, *t*(81) = 2.14, *p* = .034, *d*z = 0.25, suggesting that changes in maintenance motivation are not merely a reflection of switching to progress.

Nevertheless, goal‐shifting patterns did reveal frequent shifting from maintenance to progress. In self‐comparisons, more participants shifted to progress after downward (47%) than upward (10%) comparisons, *χ* ^2^ = 54.88, *p* < .001. In social comparisons, more shifted after upward (24%) than downward (6%) comparisons, *χ* ^2^ = 17.34, *p* < .001. To test the role of goal shifting, we added it as a mediator alongside appreciation, finding a marginally significant mediation of effects on motivation (indirect effect = .06, *p* = .059). When tested without appreciation in the model, goal shifting did significantly mediate self‐comparison effects on motivation (indirect effect = .16, 95% CI [0.08, 0.24], *p* < .001). Importantly, however, this mediation was not complete—the direct effect remained significant (direct effect = .20, *p* = .002). This suggests that goal shifting explains part, but not all, of the self‐comparison effect on maintenance motivation.

Appreciation for the current state showed the expected pattern, *F*(1, 327) = 174.99, *p* < .001, *η* ^2^ *p* = .35. Upward self‐comparisons increased appreciation (*M* = 6.32 vs. 4.70), *t*(167) = 10.36, *p* < .001, *d*z = 1.07, while upward social comparisons decreased appreciation (*M* = 5.50 vs. 6.28), *t*(160) = −8.90, *p* < .001, *d*z = 0.69. In an exploratory analysis testing appreciation as a mediator alongside goal shifting, appreciation significantly mediated self‐comparison effects (indirect effect = .34, 95% CI [0.25, 0.45], *p* < .001).

### Discussion

Study 3 extended our findings to relationships, confirming that upward self‐comparisons enhance maintenance motivation primarily through increased appreciation for one's current relationship state. The goal‐shifting analysis ruled out the alternative explanation that decreased maintenance motivation simply reflects switching to progress goals, demonstrating that maintenance represents a distinct motivational orientation beyond the absence of progress motivation.

## STUDY 4: MAINTENANCE IN EVERYDAY LIFE

Studies 1 to 3 provided strong support for our hypotheses under controlled conditions. To complement these findings with ecologically valid measures, Study 4 captured maintenance investment as it unfolds in people's everyday lives. Over 6 days, participants received twice‐daily prompts to consider either how “great” their relationship could be (upward self‐comparison) or how “terrible” it could be (downward self‐comparison). We measured participants' actual investment in maintaining their relationship since the last session and tracked how many times they engaged in their assigned comparison.

We predicted and preregistered (https://osf.io/md6fy/?view_only=6771860d44dd4a3b941365fa6abaaa9d) a two‐way interaction: the boosting effect of upward (vs. downward) self‐comparisons on maintenance investment would be stronger the more participants actually engaged with the comparison. We additionally measured appreciation and threat in each session to test whether upward self‐comparisons increase appreciation while decreasing threat. If upward self‐comparisons boost maintenance investment despite reducing threat, this would be consistent with maintenance goals operating through appreciation‐based processes rather than threat‐based protection mechanisms.

### Method

We recruited 500 UK residents in romantic relationships for over a year on Prolific. Data were collected in July 2021. We reviewed all written responses in the first session for quality as preregistered; no participants wrote nonsense content requiring exclusion. Due to a technical error, 494 participants completed the first session (73% women, *M*_age_ = 37.51, *SD* = 10.62), with completion rates for subsequent sessions ranging from 67% to 92% (*M* = 85.5%). This sample size exceeds requirements for finding stable estimates of small to moderate correlations (Schönbrodt & Perugini, 2013).

Participants were randomly assigned to upward or downward self‐comparison conditions in a 12‐session study (twice daily for 6 days) measuring maintenance investment since the last session, number of comparisons made, appreciation, and threat. In the first session, participants wrote about how they maintain their relationship and described a hypothetical scenario where their relationship was either “stronger and more harmonious than it ever was” (upward condition) or where they “drifted apart and are now more distant than you ever were” (downward condition). In each subsequent session, participants reread their maintenance text, reported their actual maintenance investment and motivation since the last session, then reread and considered their assigned comparison scenario. Finally, they reported how many times they engaged in their assigned comparison (0–10 scale), their appreciation (“Thinking about this scenario has helped me appreciate my relationship more”), and threat (“Thinking about this scenario has made me feel threatened”) since the last session on 7‐point Likert scales. Additional measures in this study and additional analyses are presented in the Supporting Information.

### Results

**FIGURE 4:**
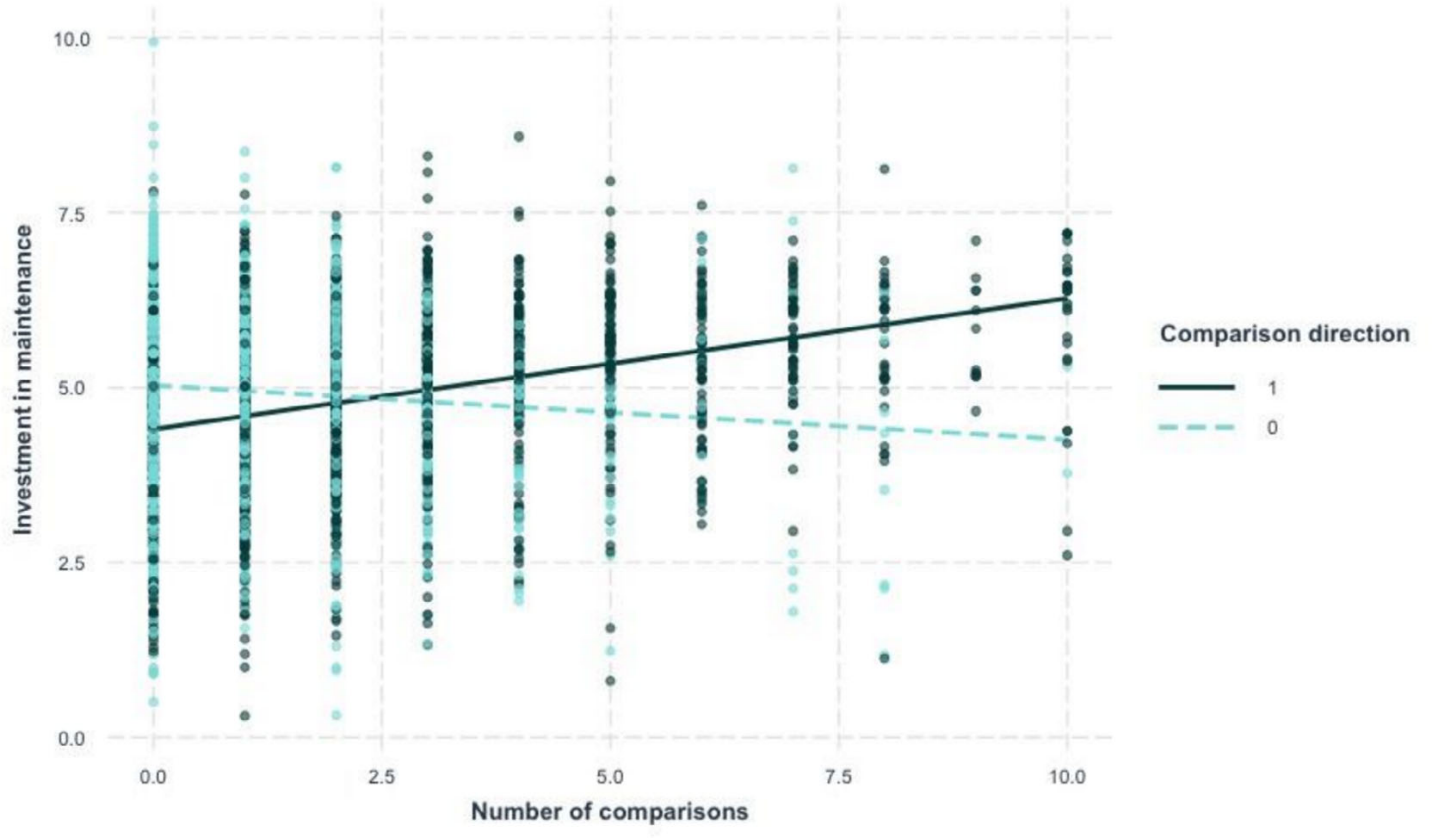
Study 4: Investment in maintenance by self‐comparison direction and number of comparisons since last session. *Note*: Comparison direction: 0 = downward, 1 = upward. Investment measured on a 7‐point Likert scale. Lines represent fitted regression slopes for each condition with all individual observations displayed.

To test our central prediction, we conducted linear mixed effects analyses using the lme4 package in R (Bates et al., 2015), with comparison direction, number of comparisons, and their interaction as fixed effects, and random intercepts for participants. *p*‐values were obtained using the lmerTest package (Kuznetsova et al., 2017). As predicted, we found an interaction of comparison direction and number of comparisons, *b* = 0.26, *SE* = 0.03, *t*(4191) = 8.70, *p* < .001, reflecting greater investment in relationship maintenance in the upward (vs. downward) condition the more comparisons were made (see Figure 4). Higher numbers of self‐comparisons predicted greater maintenance investment in the upward condition, *b* = 0.19, *SE* = 0.02, *p* < .001, and lesser investment in the downward condition, *b* = −0.08, *SE* = 0.02, *p* < .001. see Table S3 for means and correlations.

To strengthen causal inference beyond our preregistered analysis, we repeated the analysis with number of comparisons at time *t* − 1 predicting investment at time *t* while controlling for investment at time *t* − 1. This analysis found a significant interaction, *b* = 0.09, *SE* = 0.03, *t*(4185) = 3.27, *p* = .001. Using person‐centered measures of comparisons (reflecting within‐participant differences between sessions), we again found a significant interaction, *b* = 0.27, *SE* = 0.03, *t*(3710) = 8.21, *p* < .001.

Upward (vs. downward) self‐comparisons increased average appreciation, *t*(438.27) = 2.84, *p* = .005, *d* = 0.27, and decreased average threat, *t*(419.01) = −4.26, *p* < .001, *d* = −0.40. Mediation analysis revealed that appreciation at time *t* − 1 mediated the effect of comparison direction on investment in maintenance (indirect effect = .13, 95% CI [0.02, 0.24], *p* = .023), while threat did not (indirect effect = .02, 95% CI [−0.02, 0.06], *p* = .228). See Figure S1 for the indirect paths.

### Discussion

Study 4 confirmed our predictions in an ecologically valid context. Upward self‐comparisons predicted increases in maintenance investment among participants who engaged more frequently with their assigned comparison, with this effect mediated by increased appreciation for one's current relationship. Crucially, the effect occurred despite upward comparisons simultaneously decreasing threat, suggesting that maintenance motivation was driven by appreciation rather than threat‐based protection mechanisms.

The study's design allowed us to examine the potency of each comparison direction separately. Investment in maintenance correlated with comparison frequency in both upward and downward conditions, consistent with meta‐analytic evidence that both comparison directions impact motivation (Gerber et al., 2018). However, the directions of these effects differed as predicted by our theoretical framework.

Importantly, our robustness analyses address potential causal ambiguity. Because comparison frequency was measured rather than manipulated, alternative explanations based on individual differences or situational factors cannot be entirely ruled out. However, our longitudinal design provides suggestive evidence for causality: comparison frequency at time *t* − 1 predicted changes in maintenance investment at time *t*, establishing the temporal sequence required for causal inference. Moreover, person‐centered analyses examining only within‐individual changes in comparison frequency yielded similar results, excluding explanations based on stable individual differences in comparison tendencies.

## STUDY 5: BEHAVIORAL MEASURE OF MAINTENANCE

To capture maintenance motivation through actual behavior, we designed a computerized house maintenance game where participants invested effort to maintain their virtual house. We predicted and preregistered (https://aspredicted.org/ZM8_N14) that upward (vs. downward) self‐comparisons would lead to greater investment compared with the upward (vs. downward) other comparisons.

### Method

In May 2023, we recruited 1000 UK residents (ages 20–65) on Prolific (50% women, *M*_age_ = 41.83, *SD* = 12.55), providing 80% power to detect *η* ^2^ *p* = .01 as determined using G*Power (Faul et al., 2007). In the house maintenance game, which we designed for this study, participants played three rounds where they made investment decisions about maintaining their virtual house (e.g. routine roof treatment and lawn mowing; see Figure S2). In each round, participants chose their investment level: 0 points (postponing maintenance), 1 point (low quality maintenance), 2 points (medium quality), or 3 points (high quality). Critically, to “afford” higher investment levels, participants had to exert actual effort by completing a slider task—adjusting 0, 3, 6, or 9 sliders to the midpoint of the scale for 0‐, 1‐, 2‐, or 3‐point investments respectively.^2^ This slider task is a validated procedure for measuring real effort in experimental settings (Gill & Prowse, 2019).

After the first round, participants were randomly assigned to view comparisons in a 2 (comparison type: self vs. social) × 2 (comparison direction: downward vs. upward) mixed design. In the social comparison condition, participants viewed pictures and descriptions of other players' (better or worse) houses. In the self‐comparison condition, participants saw the same pictures as in the social comparison condition but were told these represented alternative versions of what their own house could have looked like. Comparisons appeared after the first and second rounds in counterbalanced order. A detailed description of all rounds in this game is included in the Supporting Information.

### Results

**FIGURE 5:**
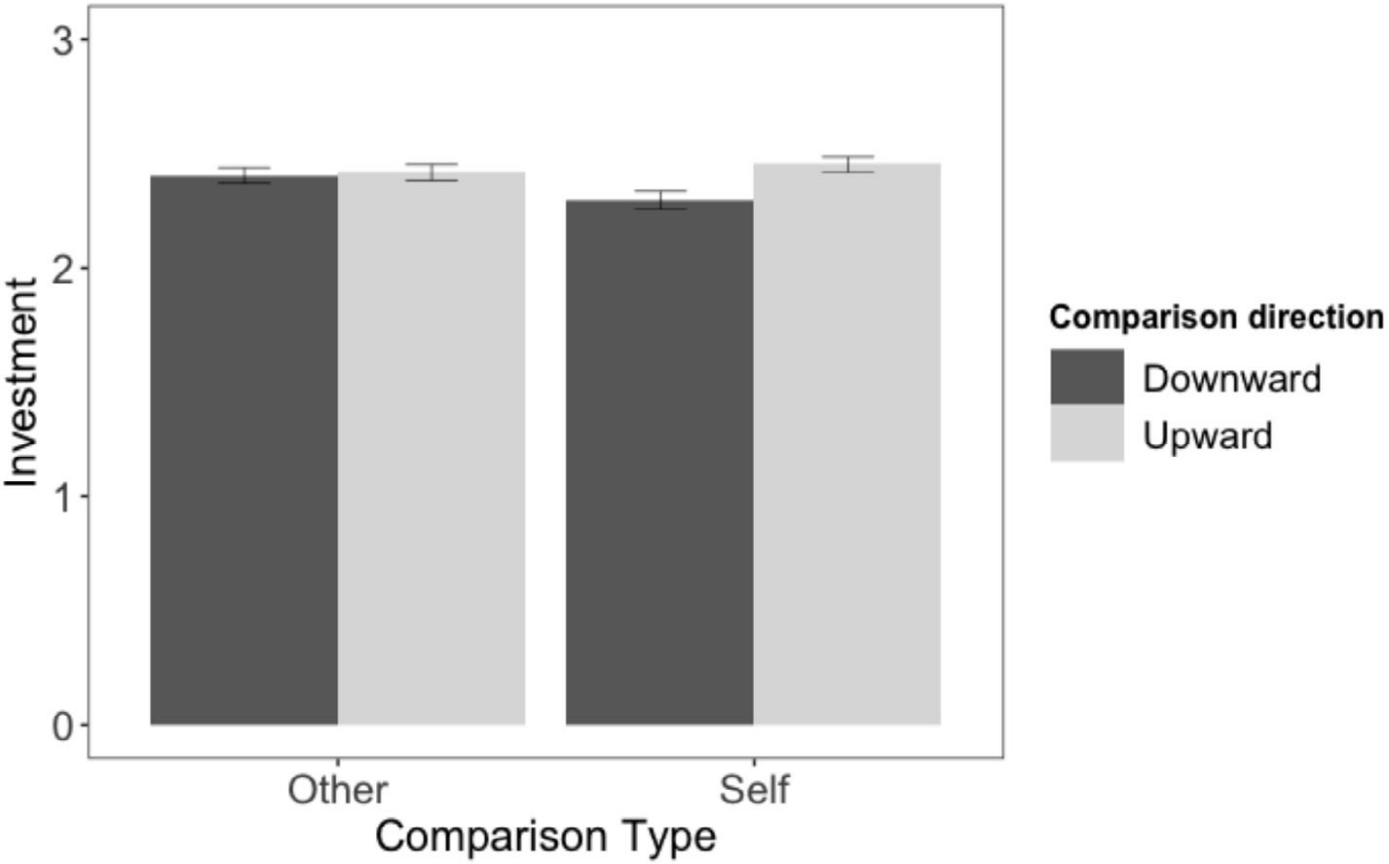
Study 5: Investment in maintaining one's house by comparison type and comparison direction. *Note*: Error bars indicate standard errors. Investment ranged between 0 and 3.

The predicted interaction emerged, *F*(1, 999) = 5.05, *p* = .025, *η* ^2^ *p* = .01. Participants invested more effort after upward self‐comparisons than downward self‐comparisons, *t*(498) = 3.18, *p* = .001, *d*z = 0.19, but showed no difference after upward versus downward social comparisons, *t*(501) = 0.29, *p* = .78, *d*z = 0.02 (see Figure 5). Additionally, upward social comparisons significantly decreased appreciation compared with downward social comparisons (*M* = 5.35 vs. 6.23), *t*(501) = 13.69, *p* < .001, but upward self‐comparisons showed no significant increase in appreciation compared with downward self‐comparisons (*M* = 5.53 vs. 5.40), *t*(498) = 1.31, *p* = .19, *d*z = 0.08. Consequently, appreciation did not significantly mediate the effect of self‐comparisons on maintenance investment in this study. See Table S4 for zero‐order correlations.

### Discussion

Study 5 demonstrated that our predictions extend to actual behavior. Participants exerted greater effort maintaining their house after considering better versions of their own house but not after viewing others' better houses. Unlike previous studies, appreciation did not mediate the self‐comparison effect on maintenance behavior. This likely reflects the greater difficulty of influencing motivation in experimental settings where goals are constructed on the spot, compared with real‐life goals rich in personal meaning. Nevertheless, the significant behavioral findings confirm that upward self‐comparisons uniquely motivate maintenance behavior beyond self‐reported measures.

## GENERAL DISCUSSION

The current research delineates the unique impact of comparisons on maintenance motivation, with critical implications for understanding and promoting health behavior maintenance. Across five studies employing diverse methodologies—experimental scenarios, experience sampling, and behavioral measures—we found evidence consistent with the hypothesis that comparison processes influence maintenance goals differently than progress goals. Study 1 provided direct evidence for this differential effect, while Studies 2–5 demonstrated that appreciation (rather than discrepancy or threat) serves as a key mediator of maintenance motivation. Studies 1 and 5 revealed that maintenance goals are not motivated by upward social comparisons, while Studies 2–5 revealed that upward self‐comparisons uniquely boost maintenance motivation. This pattern emerged across health and relationship domains, providing robust evidence for the distinct nature of maintenance motivation in contexts central to well‐being.

### Theoretical implications

Although maintenance goals are essential to personal well‐being and comprise approximately 40% of daily goal pursuit (Ebner et al., 2006; Ecker et al., 2022), they have received limited attention in psychological research (Ecker & Gilead, 2018). The predominant focus on progress goals or the binary distinction between progress and protection goals (Elliot, 2006) has led researchers to either ignore maintenance or incorrectly subsume it under protection mechanisms (Brodscholl et al., 2007). Our findings challenge these prevailing frameworks: If maintenance goals were simply a variant of protection goals, we would expect them to be motivated by threat‐related comparisons that highlight potential health risks or deterioration. Instead, we found the opposite: Maintenance motivation increased when threat decreased and appreciation increased (Studies 2–4). Similarly, if maintenance goals were merely a form of progress, upward social comparisons should have motivated them as they motivate health improvement goals (Study 1).

Maintenance should also be distinguished from “coasting”—the temporary reduction in effort when progress exceeds expectations (Carver & Scheier, 1990). Unlike coasting, which represents rest from goal pursuit, maintenance involves sustained effort to preserve valued states (Ecker, 2024). Accordingly, in Study 3, we find decreases in maintenance motivation that cannot be explained by a shift towards progress goals. Participants who experienced less appreciation for their relationship had also less motivation to invest in maintaining that relationship regardless of whether they wanted to pursue changes to the relationship. This means that, rather than “coasting,” maintenance reflects an independent type of goal investment.

While the ternary goal model (Ecker, 2024) proposes that maintenance goals operate through appreciation for one's current state rather than through discrepancy reduction, we acknowledge that maintenance behavior may sometimes involve implicit comparative elements. Traditional appraisal‐based accounts of motivation (e.g. Lazarus & Folkman, 1984) assume that effort is mobilized when there is a perceived gap or discrepancy. In some maintenance contexts, such discrepancies may exist as latent comparisons—for instance, a person maintaining their health may have a superordinate goal of “staying healthier than others my age” or may implicitly compare their current state to counterfactual “what if” scenarios of decline. Such comparative elements represent additional motivational layers rather than the core appreciation‐based mechanism driving day‐to‐day maintenance behavior. This distinction between core maintenance mechanisms (appreciation driven) and potential superordinate comparative goals (discrepancy driven) merits further theoretical and empirical attention in future research.

### Practical implications for health interventions

Understanding maintenance motivation offers a pathway to more effective health behavior interventions. Social comparison is widely employed as a behavior change technique in health interventions across diverse contexts (Hoppen et al., 2025). However, our findings suggest that maintenance‐focused interventions should fundamentally differ from change‐focused approaches. Rather than highlighting others' superior health outcomes, effective maintenance interventions should emphasize self‐comparison strategies that enhance appreciation for current health achievements. Interestingly, while social comparison research has shown that self‐enhancement (feeling good) and self‐improvement (doing more) motives are typically served by different comparison directions—with downward comparisons boosting self‐esteem and upward comparisons facilitating goal pursuit (Morina, 2021; Taylor & Lobel, 1989; Wills, 1981), our findings reveal a context where the two motives align: when it comes to maintenance, upward self‐comparisons simultaneously enhance appreciation for one's current state and commitment to preserving it.

For health practitioners and intervention designers, this research provides concrete guidance: When working with patients who have successfully adopted healthy behaviors (regular exercise, healthy eating, and medication adherence), avoid comparisons with healthier others and instead encourage reflection on personal progress and current health benefits. Health apps and fitness trackers could be redesigned to emphasize personal milestones and current state appreciation rather than social rankings or comparisons to fitter users. Workplace wellness programs could shift from highlighting top performers to helping employees appreciate their current health. This approach is particularly crucial for chronic disease management, where ongoing self‐care behaviors are essential but offer no promise of improvement—only the maintenance of current functioning. For patients managing diabetes, hypertension, or other chronic conditions, maintenance motivation may be more critical than change motivation.

### Limitations and future directions

#### The role of regulatory focus

Regulatory focus theory distinguishes between promotion focus (concerned with growth, advancement, and positive outcomes) and prevention focus (concerned with safety, security, and the lack of negative outcomes; Higgins, 1997). Our research kept a consistent promotion focus across studies (e.g. “maintaining (or not maintaining) current health” rather than “maintaining (or not maintaining) safety from illness”), ensuring that goal type rather than regulatory focus drove our observed effects. Future psychology research should examine whether prevention‐focused maintenance goals respond differently to comparison processes, as this could inform interventions for different patient populations.

#### Comparing with protection goals

Protection goals are activated in response to threats and aim to reduce or eliminate anticipated negative outcomes (Ecker, 2024). Unlike maintenance goals, which involve ongoing care in stable conditions, protection goals tend to be shorter term, reactive responses that culminate once the threat is removed. For instance, when one worries about contracting the flu, they may get vaccinated to reduce this threat—a discrete protective action rather than ongoing maintenance behavior. Because protection goals tend to be more concrete and time‐limited than maintenance and progress goals, they present unique methodological challenges for experimental comparison. While we refrained from direct comparisons in this research, future studies should examine how comparisons influence protection motivation. Consistent with the ternary goal model (Ecker, 2024) and protection motivation theory (Rogers, 1983), we predict that threatening comparisons would motivate protection goals, contrasting sharply with maintenance goals' responsiveness to appreciation‐enhancing comparisons. Such research could further validate the ternary framework by demonstrating distinct comparison effects across all three goal types.

#### Generalizability concerns

Our research focused on providing proof‐of‐concept evidence for distinct comparison effects on maintenance goals using samples from English‐speaking countries. Given cultural differences in approach‐avoidance dynamics (Elliot et al., 2001; Hamamura et al., 2009) and some evidence for cultural variation in maintenance pursuit (Yang et al., 2015), testing these effects across diverse cultural contexts represents an important next step for establishing the universality of these mechanisms.

Finally, our studies compared upward and downward conditions without a neutral control. This was deliberate: A “neutral” comparison (e.g. to someone similar or no comparison) would introduce confounds such as feelings of comradery or social presence (Briskin et al., 2019; vanDellen et al., 2015). Future research could nevertheless explore whether effects are driven more by upward or downward comparisons.

## CONCLUSION

This research provides both theoretical insight and practical tools for addressing the maintenance challenge. Five studies—combining experimental scenarios, experience sampling, and behavioral measures—converge in showing that upward self‐comparisons enhance maintenance motivation through increased appreciation for current states, while upward social comparisons that effectively motivate progress goals do not motivate maintenance goals in the same way. These findings challenge traditional binary frameworks of motivation and provide compelling evidence for maintenance as a distinct goal type with unique mechanisms. Rather than relying on social comparison strategies that work for behavior change, health interventions focused on maintenance should emphasize appreciation for current health. This research offers evidence‐based strategies for supporting long‐term health behavior maintenance and addresses one of health psychology's most persistent challenges.

## CONFLICT OF INTEREST STATEMENT

The authors declare no conflict of interest.

## ETHICS STATEMENT

All procedures were conducted in accord with the American Psychological Association Ethical Principles of Psychologists and Code of Conduct (APA, 2017). At the senior author's institution (JGU Mainz, Department of Psychology, Ethics Committee), studies that did not involve deception, vulnerable populations, identifiable data, intensive data, or interventions were exempt from ethical approval and not evaluated at time of data collection.

## Supporting information


**Table S1.** Study S1: Zero order correlations between motivation, threat, and appreciation for the current state by comparison type and comparison direction.
**Table S2.** Study 3: Zero order correlations between motivation, threat, appreciation for the current state, and goal shifts by comparison type and comparison direction.
**Table S3.** Study 4: Mean scores and standard deviations on all variables by comparison direction condition.
**Figure S1.** Indirect Paths via Appreciation and Threat in the Impact of Upward (vs. Downward) Comparisons on Investment in Maintenance. Mediators Co‐Varied with the DV both Between (Level 2) and Within (Level 1) Participants.
**Figure S2.** Participants' House as Presented in All Experimental Conditions in the House Maintenance Game.
**Table S4.** Study 5: Zero order correlations between motivation, appreciation, and threat by goal type and comparison type condition.

## Data Availability

The materials and the data are available at https://osf.io/bys5t/.

## ENDNOTES

1 We also measured perceived attainability and global value of the health domain as preregistered variables pertaining to additional hypotheses that go beyond the scope of the current paper. Complete results and interpretation are reported in the Supporting Information.

2 The preregistration mistakenly stated 12 sliders for the highest level; the actual study used the progression of 0, 3, 6, and 9 sliders as described here.
