# Supplementary material for: The role of comparison processes in maintenance goals: Evidence from the health and relationship domains
Source: Appl Psychol Health Well Being. 2026 Mar 10;18(2):e70133. doi: 10.1111/aphw.70133 (PMC12973158; doi:10.1111/aphw.70133)
Supplement: Supplementary file 1 — Table S1. Study S1: Zero order correlations between motivation, threat, and appreciation for the current state by comparison type and comparison direction. Table S2. Study 3: Zero order correlations between motivation, threat, appreciation for the current state, and goal shifts by comparison type and comparison direction. Table S3. Study 4: Mean scores and standard deviations on all variables by comparison direction condition. Figure S1. Indirect Paths via Appreciation and Threat in the Impact of Upward (vs. Downward) Comparisons on Investment in Maintenance. Mediators Co‐Varied with the DV both Between (Level 2) and Within (Level 1) Participants. Figure S2. Participants' House as Presented in All Experimental Conditions in the House Maintenance Game. Table S4. Study 5: Zero order correlations between motivation, appreciation, and threat by goal type and comparison type condition. [file APHW-18-0-s001.docx]

**Supplementary Material**

**Study 1**

***Full Procedure***

Participants were randomly assigned to an approach or a maintenance goal type condition. Accordingly, we asked participants to report either a health improvement goal (“*aspects of your physical health that you are unsatisfied with, and that require your attention and care to be improved*”) or a health maintenance goal (“*aspects of your physical health that you appreciate, and that require your attention and care to be maintained*”). We then introduced all participants with a downward and an upward health standard in randomized order. In the upward standard condition, participants read about the following new neighbors: *“…they are healthier and in better shape than you are. They like to do athletic activities in their free time and to cook nutritious food at home. They have mentioned to you once or twice that they used to have a very unhealthy lifestyle but they have since completely transformed and enjoy a lifestyle that enables them to remain in perfect health*”. In the downward standard condition, participants read about these neighbors: *“…they are less healthy and in worse shape than you are. They don't like to move much in their free time and they rely mostly on unhealthy and fast food. They have mentioned to you once or twice that they used to have a healthier lifestyle but they have since completely transformed to a lifestyle that makes them sick often and generally unhealthy.*”

After considering each scenario, participants reported their agreement with four statements (on a 1-7 scale): two statements measuring motivation, as well as a statement measuring appreciation for the current state and a statement measuring threat. Additionally, we measured perceptions of attainability (“*Thinking about this scenario makes me feel that my chances to succeed in maintaining/improving my current health are:*”; scale ranged from “*1 – Very low*” to “*7 – Very high*”) and global value of the health domain (“*Thinking about this scenario makes me feel that personal health in general is:*”; scale from “*1 – Not really so important*” to “*7 – The most important assent one can have*”), which we report in the supplementary material. At the end of the study, participants reported their age and gender as in Study 1, as well as self-evaluated fitness and health: “*How would you rate your fitness level?*”, “*How would you rate your current health?*” (1 – “*much below average*”, 5 – “*much above average*”). Results pertaining to these measures are also reported in the supplementary material.

***The Experience of Threat***

A 2 (goal type: maintenance, approach; between participants) x 2 (comparison direction: downward, upward; within participants) ANOVA on general threat about one’s health found a robust main effect of comparison direction, *F*(1, 400) = 161.18, *p* < .001, $\eta_{p}^{2}$ = 0.29, reflecting a greater experience of threat after *upward* (*M* = 2.98, *SD* = 1.77) compared to downward (*M* = 1.84, *SD* = 1.23) social comparisons.

***Global Value of Health Domain***

Global value was lower after upward comparisons (*M* = 5.58, 5.69, *SD* = 1.14, 0.99) than downward comparisons (*M* = 5.69, 5.84, *SD* = 1.03, 1.01; *d* = 0.13, 0.18, *p* = .054, .011) in both the approach (albeit marginal significance) and maintenance goal conditions, respectively.

***Attainability of Goal***

Attainability was similar after upward comparisons (*M* = 4.94, 5.20, *SD* = 1.26, 1.24) and downward comparisons (*M* = 4.89, 5.33, *SD* = 1.18, 1.16; *d* = 0.04, 0.11, *p* = .583, .118) in both the approach and maintenance goal conditions, respectively.

**Study 2**

***Full* *Procedure***

At the beginning of the study, participants read the following text: “*In this study, we are interested in your health maintenance goals. These are aspects of your physical health that require your attention and care to be maintained. Please name below two central activities that you regularly do to maintain health*”. After participants reported two health maintenance activities and confirmed that they indeed view these activities as maintenance, all participants viewed a downward and an upward comparison in random order. For half of the participants, these were other-comparisons while for the rest they were self-comparisons.

In the other-comparison condition, participants were asked to imagine having new neighbors which are either very healthy (“*You notice that they are healthier than you are. They always seem energetic and they rarely get sick. Every morning they wake up early full of energy to start the day and in the evenings, they still seem refreshed and ready to spend their free time doing the things they like.*”) or very unhealthy (“*You notice that they are less healthy than you are. They are often sick for weeks at a time and rarely seem to be in good health. Every morning they wake up tired, often with a cough or other cold symptoms, dragging their feet out of the house, and in the evenings, they seem completely exhausted and ready to spend all their free time in bed or on the sofa.*”). In the self-comparison conditions, participants were asked to imagine similar versions of healthiness and unhealthiness about themselves (e.g., “*You notice that you are healthier than you ever were. You are always energetic and you rarely get sick. Every morning you wake up early full of energy to start the day and in the evenings, you still feel refreshed and ready to spend your free time doing the things you like.*”).

After viewing each comparison, participants indicated their agreement with a set of statements on a 7-point scale: “After reading this scenario, I feel...” – “*appreciation for my current level of health*”. – “*threatened about my current level of health*”. – “*motivated to maintain my current health by engaging in the activities I named before*”. – “*encouraged to maintain my current health by putting effort in the activities I named before*”. These items measured appreciation, threat, and motivation (as average of two items), respectively. Table S1 show zero-order correlations between all variables in all experimental conditions.

***Table S1***

*Study S1: Zero order correlations between motivation, threat, and appreciation for the current state by comparison type and comparison direction.*

| Comparison Type |  | Downward comparisons | | | Upward comparisons | | |
| --- | --- | --- | --- | --- | --- | --- | --- |
|  |  | Motivation | | Appreciation | Motivation | | Appreciation |
| *Self* | Motivation |  |  | |  |  | |
|  | Appreciation | .83*** |  | | .75*** |  | |
|  | Threat | -.40*** | -.48*** | | -.32*** | -.44*** | |
| *Other* | Motivation |  |  | |  |  | |
|  | Appreciation | .60*** |  | | .53*** |  | |
|  | Threat | .09 | -.17 | | -.03 | -.21* | |

*Notes*. * indicates a significant correlation with *p* < . 05; ** *p* < .01; *** *p* < .001.

**Study 3**

***Full Procedure***

At the beginning of the study, participants read the following text: “*In this study, we are interested in your relationship maintenance goals. These are aspects of your relationship that require your attention and care to be maintained.* *Please name below two central aspects that you try to maintain in your relationship*”. After reporting the two aspects of their relationship they wish to maintain, and indicating whether they wish to maintain or improve them, participants were randomly assigned to a self- or other- comparison type condition.

In the other-comparison condition, participants viewed neighbors with a worse and better relationship in randomized order. The better off neighbors were described as follows: “…*the relationship between the two partners is stronger and more harmonious than the one between you and your partner. They clearly love each other, have a lot of fun together, and feel satisfied in their relationship*”. The worse off neighbors were described as follows: “…*the relationship between the two partners is not good. They fight considerably more than you and your partner do, and seem to generally treat each other coldly and with some hostility*”. In the self-comparison condition participants viewed similar texts that were introduced as possible versions of their own relationship.

After viewing each comparison standard, participants rated their agreement with statements measuring appreciation, threat, and motivation. In measuring threat, our intention was to test whether a potential motivational boost correlates with increases in threat. Because this was very clearly not the case, considerations of brevity led us to report analyses of scores on this item in the supplements. Additionally, participants were asked after each comparison standard whether their maintenance goal shifted to approach: “*After reading this scenario, which of the options below best describes how you view the aspects of your relationship you listed before?*” – “*I still want to maintain these aspects*”. – “*I now prefer to improve these aspects*”. – “*Neither*”.

***Table S2***

*Study 3: Zero order correlations between motivation, threat, appreciation for the current state, and goal shifts by comparison type and comparison direction.*

| Comp. Type |  | Downward comparisons | | | | Upward comparisons | | | |
| --- | --- | --- | --- | --- | --- | --- | --- | --- | --- |
|  |  | Motivation | Appreciation | | Threat | | Motivation | Appreciation | Threat |
| *Self* | Motivation |  | |  |  | |  |  |  |
|  | Appreciation | .63*** | |  |  | | .54*** |  |  |
|  | Threat | -.31*** | | -.59*** |  | | -.32*** | -.42*** |  |
|  | Goal shifts | -.34*** | | -.33*** | .35*** | | .11 | -.01 | .09 |
| *Other* | Motivation |  | |  |  | |  |  |  |
|  | Appreciation | .51*** | |  |  | | .47*** |  |  |
|  | Threat | -.31*** | | -.46*** |  | | .08 | -.40*** |  |
|  | Goal shifts | .08 | | .06 | -.11 | | .19* | -.15 | .28*** |

*Notes*. * indicates a significant correlation with *p* < . 05; ** *p* < .01; *** *p* < .001.

**Study 4**

***Full Procedure***

The first session was conducted on a Monday morning. All five subsequent morning sessions (Tuesday to Saturday) were open to all participants who took part in the first session, from 6:00 to 12:00 AM. Additionally, five evening sessions (Monday to Friday) were open from 5 PM until midnight. The first session opened with a five-item standard measure of relationship satisfaction (Rusbult, et al., 1998). We then instructed participants to write a text describing the following: “*1. what you ideally would do daily to maintain your relationship. 2. what you actually do daily to maintain your relationship. 3. and what you intend to do in the coming week to maintain your relationship*”. After the writing task, participants reported their baseline motivation: “*How motivated are you to maintain your relationship this morning?*” and “*How willing are you to put the effort required to maintain your relationship this morning?*”.

On all subsequent (evening and morning) measurements, the first part of the session included a measure of actual maintenance investment and a two-item measure of maintenance motivation. Actual investment was measured as follows: “*Think about the efforts you make and wish to make to maintain your relationship:”* (piped text from first session). “*Now think specifically about (today/ this morning and yesterday evening). Since the last study session (this morning/ yesterday evening), how much time, effort, or resources did you invest into your relationship with your partner?*” (1- *None*, 7- *Very much*). Motivation was measured as follows: “*Since the last study session (yesterday evening/ this morning)*” – (1) “*How motivated have you been to maintain your relationship?*” (2) “*How willing have you been to put the effort required to maintain your relationship?”* (1 - *Not at all or extremely little*, 7 – *Extremely so*).

The second part of each session included a manipulation of self-comparison direction. In the first session, participants were instructed to write a short text about a hypothetical situation where they notice that their relationship with their partner is “*stronger and more harmonious than it ever was*” (upward comparison) or that they “*drifted apart and are now more distant than you ever were*” (downward comparison). We further instructed all participants to formulate “*how you envision this scenario to be. For instance, what in particular (would your disagreements be about/ would be fun to do together) and what would your life look like when feeling so (distant/ close)*”. Subsequent sessions did not include a writing task; We only asked participants to consider the scenario in their respective comparison condition, and piped back the text they wrote in the first session. Importantly, at the end of each session, we requested participants to “*try to keep in mind the scenario you wrote about, and envision how great (terrible) your relationship could have been*”.

After the comparison direction manipulation, on all but the first session, we asked participants how much they engaged in the comparison in their respective condition: “*Since the last study session (yesterday evening/ this morning) - How many times did you find yourself thinking about this or a similar scenario where your relationship is much (worse/ better)?*” (0-10). Finally, we asked participants to report appreciation and threat since the last session: (1) “*Thinking about this scenario has helped me appreciate my relationship more”.* (Appreciation; 1-7) (2) “*Thinking about this scenario has made me feel threatened*”. (Threat; 1-7). The baseline measurements of appreciation and threat in the first session were similar, without the instruction to think back up to the last session.

***Additional Analyses***

**Main effects of comparison direction on investment*.*** The comparison direction manipulation did not affect investment in relationship maintenance, *t*(441.99) = 1.12, *p* = .263, *d* = 0.11, nor self-reported relationship maintenance motivation throughout the measurement period, *t*(441.98) = 0.65, *p* = .514, *d* = 0.06. Upward (vs. downward) self-comparisons did, however, increase appreciation for current state of the relationship, as measured at the first session, *t*(428.22) = 3.47, *p* < .001, *d* = 0.33, as well as the averaged appreciation in subsequent sessions, *t*(438.27) = 2.84, *p* = .005, *d* = 0.27. Additionally, upward self-comparisons decreased threat at the first session, *t*(394.3) = 7.77, *p* < .001, *d* = 0.73, and average threat in subsequent sessions, *t*(419.01) = -4.26, *p* < .001 , *d* = -0.40. This is not surprising, because participants reported threat and appreciation immediately after the manipulation in each session, while motivation and investment were reported in each session just before the manipulation. Therefore, only the appreciation and threat measures benefited from the manipulation’s immediate influence, while motivation and investment could benefit only from potential effects of the previous sessions.

**Baseline Compared to Subsequent Motivation**. In our pre-registration, we predicted that overall self-reported motivation in the upward comparison condition will be higher than the baseline motivation reported in the first session. In fact, the results show that average subsequent motivation was lower compared to baseline in both the upward, *t*(219) = 6.22, *p* < .001 , *d* = 0.36, and downward comparison condition, *t*(223) = 5.25, *p* < .001, *d* = 0.32. This was likely due to a somewhat faulty baseline measurement – while subsequent measurements referred to motivation within a specific time frame in the past (since last measurement, either that morning or evening of previous day), the baseline measurement referred to how participants felt right now, during the completion of the study. It seems reasonable to assume that this difference in the time frame may have changed the nature of the reported construct. A report of current motivation is likely strongly based on an internal experience, whereas motivation during a specific time period in the past can rely instead on memory of overt behavior.

***Table S3***

*Study 4: Mean scores and standard deviations on all variables by comparison direction condition.*

| Measure | Comparison direction | | Correlations | | | | | | | | |
| --- | --- | --- | --- | --- | --- | --- | --- | --- | --- | --- | --- |
|  | **Down**  *M (SD)* | **Up**  *M (SD)* | 1 | 2 | 3 | 4 | 5 | 6 | 7 | 8 | 9 |
| *1. Satisfaction 1^st^ session* | 5.768 (1.21) ^A^ | 5.730 (1.20) ^A^ |  |  |  |  |  |  |  |  |  |
| *2. Satisfaction last session* | 5.776 (1.32) ^A^ | 5.722 (1.26) ^A^ | .84*** |  |  |  |  |  |  |  |  |
| *3. Motivation 1^st^ session* | 5.868 (1.28) ^A^ | 5.832 (1.22) ^A^ | .71*** | .66*** |  |  |  |  |  |  |  |
| *4. Motivation all sessions.* | 5.474 (1.19) ^A^ | 5.401 (1.18) ^A^ | .56*** | .65*** | .63*** |  |  |  |  |  |  |
| *5. Appreciation 1^st^ session* | 5.156 (1.82) ^A^ | 5.705 (1.49) ^B^ | .61*** | .63*** | .54*** | .48*** |  |  |  |  |  |
| *6. Appreciation all sessions.* | 4.139 (1.80) ^A^ | 4.598 (1.61) ^B^ | .29*** | .39*** | .30*** | .53*** | .42*** |  |  |  |  |
| *7. Threat 1^st^ session.* | 2.879 (1.76) ^A^ | 1.768 (1.20) ^B^ | -.18*** | -.19*** | -.12** | -.12** | -.34*** | -.08 |  |  |  |
| *8. Threat all sessions.* | 2.033 (1.26) ^A^ | 1.578 (0.97) ^B^ | -.12** | -.13** | -.07 | .04 | -.17*** | .08 | .55*** |  |  |
| *9. Investment in maintenance all sessions.* | 4.943 (1.2) ^A^ | 4.813 (1.21) ^A^ | .48*** | .52*** | .51*** | .84*** | .40*** | .51*** | -.08 | .02 |  |
| *10. Commitment 1^st^ session* | 6.290 (0.85) ^A^ | 6.289 (0.89) ^A^ | .67*** | .81*** | .52*** | .54*** | .52*** | .33*** | -.18*** | -.18*** | .41*** |

*Notes*. * indicates a significant correlation with *p* < . 05; ** *p* < .01; *** *p* < .001. Level 1 variables were transformed to Level 2 by averaging across sessions. Different superscripts in one row indicate a significant difference between the two values. The response scales on all measures ranged from 1 to 7.

***Appreciation and Threat***

As predicted, upward (vs. downward) self-comparisons increased participants’ average appreciation for their relationship across all sessions, *t*(438.27) = 2.84, *p* = .005, *d* = 0.27, and decreased average threat across sessions, *t*(419.01) = -4.26, *p* < .001 , *d* = -0.40. We used the MLMED macro for SPSS (Hayes & Rockwood, 2020) to calculate a 2-1-1 mediation, with comparison direction as a Level 2 predictor, appreciation and threat at time t-1 as Level 1 mediators, and investment in maintenance as the dependent variable. We also entered investment at time t-1 as a co-variate. As Figure S1 shows, we found an indirect effect whereby appreciation at time t-1 mediated the effect of comparison direction on investment in maintenance: *indirect effect* = 0.13, *SE* = 0.06, 95% *CI* [0.02, 0.24], *z* = 2.27, *p* = .023. A similar indirect effect was not significant for mediation by threat at time t-1: *indirect effect* = 0.02, *SE* = 0.02, 95% *CI* [-0.02, 0.06], *z* = 1.20, *p* = .228.

***Figure S1***

*Indirect Paths via Appreciation and Threat in the Impact of Upward (vs. Downward) Comparisons on Investment in Maintenance. Mediators Co-Varied with the DV both Between (Level 2) and Within (Level 1) Participants.*


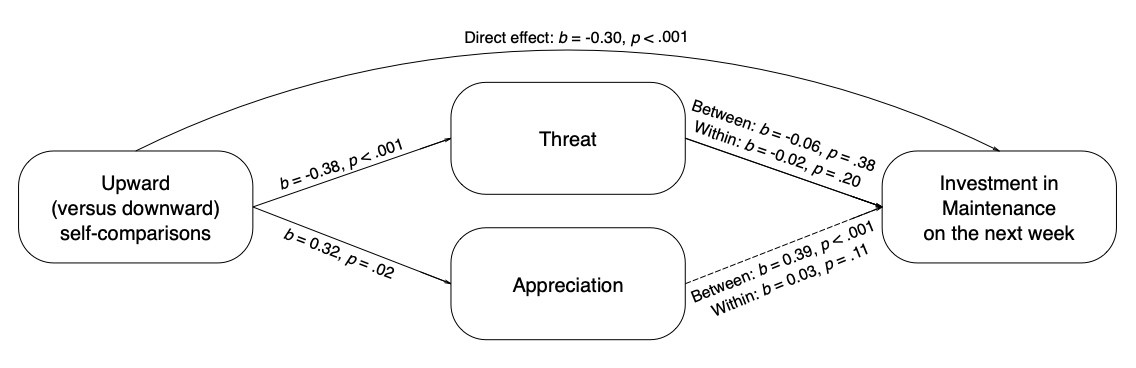


**Study 5**

***Full Procedure***

At the beginning of the study, participants received the following instructions:

“*In each round of this game, you will decide how much to invest in the different services needed for maintaining a house. You will face five decisions about the quality of construction material and the contractors you hire, choosing either low, average, or high quality:*

- *Postponing maintenance work (investing 0 points) - Making this decision will not require any extra effort from you.*
- *Low quality (investing 1 point) - this will require you to invest a little bit of time in "earning" the point you chose to invest. Specifically, we will ask you to set 3 sliders to the middle of the scale in order to earn your point.*
- *Average quality (investing 2 point) - this will require you to set 6 sliders to the middle of the scale in order earn your points.*
- *High quality (investing 3 points) - this will require you to set 9 sliders to the middle of the scale in order to earn your points.”*

After reading the instructions, participants adjusted a few example sliders and were then presented with house they will maintain. The first maintenance round concerned the roof of the house: “*Winter snow and ice damage shingles which could lead to leaks. Your roof must be inspected and routine maintenance work by a qualified contractor is required*. *How much are you willing to invest in maintaining your house and keeping it in good shape?*”. Participants could choose between investing 0 points (postponing maintenance), 1 point (low quality), 2 points (medium quality), and 3 points (high quality). They were explained that a 0-point investment meant not adjusting any sliders, whereas 1-, 2-, and 3-point investments meant adjusting 3, 6, or 9 sliders respectively.

***Figure S2***

*Participants’ House as Presented in All Experimental Conditions in the House Maintenance Game.*


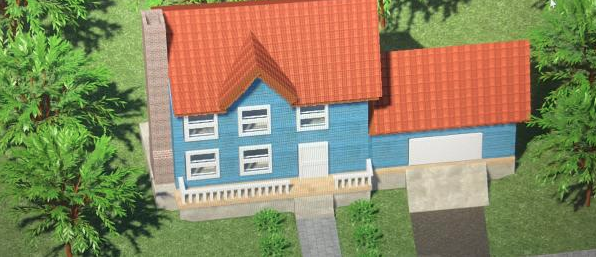


After the first round, participants were randomly allocated to a self or other comparison type condition (between-participants). Each participant viewed both a downward and an upward standard (within-participants), one appearing after the first round and the other after the second round (in counterbalanced order). In both comparison type conditions participants saw the same pictures of good- and bad-looking houses. In the other comparison condition, participants read that other participants’ houses are different from theirs. The downward standard condition described these houses as “*shaped like a matchbox, dilapidated, utterly unappealing and underwhelming, with scant space and tiny windows, and outside, no patio nor a garden - in short, awful homes*”. The upward standard described the other houses as having “*a grand and gleaming entrance door, poised to impress, spacious rooms with large windows, and Outside, a beautiful patio and a stunning garden - in short, gorgeous homes*”. In the self-comparison condition, participants read similar descriptions but were asked to consider them as alternative versions of what their house could have looked like. After each comparison standard, participants answered one question on a 7-point scale: “*Thinking about this house, how much do you appreciate the house you are maintaining in the game?*” They then moved on to the second or third round of the game, similarly structured as round 1, this time concerning the house’s foundation and HVAC, respectively.

After each round, participants indicated their level of investment between 0 and 3 points. They then continued to set the corresponding number of scale pointers to the midpoint of the scale: none when investing 0 point and 3, 6, and 9 sliders when investing 1, 2, and 3 points, respectively. This measure follows the *slider task* - a known validated procedure for measuring real effort in experimental settings (Gill & Prowse, 2019).

***Table S4***

*Study 5: Zero order correlations between motivation, appreciation, and threat by goal type and comparison type condition.*

| Comparison Type | Comparison direction | Investment-Appreciation correlation |
| --- | --- | --- |
| *Self-comparisons* | ***Upward*** | 0.31*** |
|  | ***Downward*** | 0.26*** |
| *Other-comparisons* | ***Upward*** | 0.20*** |
|  | ***Downward*** | 0.31*** |

*Notes*. * indicates a significant correlation with *p* < . 05; ** *p* < .01; *** *p* < .001.

**Scenario Validation**

Although we did not conduct separate pretests, the scenarios were validated through embedded manipulation checks and cross-study replication. Across all studies, upward social comparisons decreased appreciation while upward self-comparisons increased appreciation, confirming the scenarios produced the intended psychological states. The consistent pattern of results across different scenarios in health, relationship, and household domains provides convergent evidence that findings reflect the theoretical constructs rather than idiosyncratic scenario features.
